# Case of Fracture of the Skull and Wound of the Brain

**Published:** 1858-01

**Authors:** Ellerslie Wallace

**Affiliations:** Demonstrator of Anatomy in the Jefferson Medical College of Philadelphia


ART. VI.—*Case of Fracture of the Skull and Injury of the Brain by a Circular Saw.* By ELLERSLIE WALLACE, M.D., Demonstrator of Anatomy in the Jefferson Medical College of Philadelphia.

I WAS called on the morning of the 31st of July to a little girl ten years of age, who, in stooping down under a circular saw of $14\frac{1}{4}$ inches diameter, which was revolving two thousand times per minute, received from the saw a wound of the following description:—

It passed transversely across the head, nearly coinciding with the line of the coronal suture; its length was $4\frac{1}{3}$ inches through the scalp, and precisely the same through the bone. The width of the gap in the bone was one-sixth of an inch. The greatest depth of the wound was at its middle, in the line of the sagittal suture, at which point it extended vertically down into the cavity of the cranium, one inch and an eighth from the outer surface of the scalp.

Of course the superior longitudinal sinus was divided; whether the falx cerebri was entirely cut through or not I am not certain, but from the size and shape of the head I think it fairly possible that such was the case; in such event, the inferior longitudinal sinus, also, must of necessity have been divided.

The edges of the scalp-wound had retracted about a quarter of an inch from the margins of the cut through the bone, and the divided surfaces were as clean and smooth as if the section had been made with a sharp scalpel. As far as could be seen, there was no foreign matter in the wound, nor any granules from the bone; the great speed of the saw had cleared its track completely. I did not see the patient until more than an hour after the infliction of the injury. I found her then pallid, with a cool skin and feeble pulse. Her intellect was unclouded, and she expressed herself as free from any pain or distress, except a little soreness about the wound. The hemorrhage had been profuse and exhausting, but it had now ceased, and the aperture in the bone was nearly filled with clot, a portion of which extended between the edges of the scalp for a short space.

I directed the hair to be carefully shaved from the vicinity of the injury, and then laid her in bed on her side, to favor the discharge of any blood that might yet be effused into the cavity of the cranium; I did not draw the soft tissues together at all, but merely laid a linen rag, dipped in cool water, over the front half of the head, directing it to be changed when it should become warm. Sago and cold water were allowed as diet, and perfect quietude enjoined. She slept occasionally during the day, and by evening had rallied somewhat, and there was no return of hemorrhage.

Until the lapse of three days the treatment was not in any respect changed; for she continued entirely comfortable, and while her reaction was perfect there had not been any evidence of the slightest disorder of her system.

The edges of the scalp were then approximated by two adhesive strips, but were not completely drawn together, as I did not deem it prudent to cover the wound in the skull by perfect coaptation of the soft tissues. On the sixth day, as every point in her case was favorable, I allowed her broths, with full farinaceous diet. During the second week granulations from the cut surfaces of the scalp filled up the space that had been allowed to remain between the edges, and meat was now permitted her, with the usual vegetables in season. She was dressed and sat up in her room in the third week, and under simple dressings the wound went on regularly and favorably to cicatrization, though the process was somewhat slow and tedious.

Between the seat of injury and the vertex of the head there was no sensation for some time after the cicatrix had formed, but on the 15th October I found that all feeling had returned.

During the entire progress of the case the child never suffered pain, sickness, fever, loss of sleep, or loss of appetite.

Upon two occasions only was it needful to administer a cathartic, to obviate a tendency to constipation.

Her mental faculties are now as perfect as ever, and her physical condition is more vigorous than before the accident.

Dr. Eaton assisted me in attending her for several days, and during my absence from the city for a time, Dr. Hoyt had charge of her for me, and from him I obtained some of the particulars above recorded.

